# Terrestrial acidification and ecosystem services: effects of acid rain on bunnies, baseball, and Christmas trees

**DOI:** 10.1002/ecs2.1857

**Published:** 2017-06

**Authors:** IRINA C. IRVINE, TARA GREAVER, JENNIFER PHELAN, ROBERT D. SABO, GEORGE VAN HOUTVEN

**Affiliations:** 1 Santa Monica Mountains National Recreation Area, Division of Planning Science and Resource Management, US National Park Service, Thousand Oaks, California 91360 USA; 2 Office of Research and Development, National Center for Environmental Assessment, US Environmental Protection Agency, Research Triangle Park, North Carolina 27709 USA; 3 RTI International, Research Triangle Park, North Carolina 27709 USA; 4 Office of Research and Development, National Climate Assessment Global Change Impacts and Adaptations, Environmental Protection Agency, Crystal City, Virginia 22202 USA

**Keywords:** acid deposition, balsam fir, critical load, ecosystem services, forests, nitrogen, Special Feature: Air Quality and Ecosystem Services, sulfur, white ash

## Abstract

Often termed “acid rain,” combined nitrogen and sulfur deposition can directly and indirectly impact the condition and health of forest ecosystems. Researchers use critical loads (CLs) to describe response thresholds, and recent studies on acid-sensitive biological indicators show that forests continue to be at risk from terrestrial acidification. However, rarely are impacts translated into changes in “ecosystem services” that impact human well-being. Further, the relevance of this research to the general public is seldom communicated in terms that can motivate action to protect valuable resources. To understand how changes in biological indicators affect human well-being, we used the STEPS (Stressor–Ecological Production function–final ecosystem Services) Framework to quantitatively and qualitatively link CL exceedances to ecosystem service impacts. We specified the cause-and-effect ecological processes linking changes in biological indicators to final ecosystem services. The Final Ecosystem Goods and Services Classification System (FEGS-CS) was used within the STEPS Framework to classify the ecosystem component and the beneficiary class that uses or values the component. We analyzed two acid-sensitive tree species, balsam fir (*Abies balsamea*) and white ash (*Fraxinus americana*), that are common in northeastern USA. These well-known species provide habitat for animals and popular forest products that are relatable to a broad audience. We identified 160 chains with 10 classes of human beneficiaries for balsam fir and white ash combined, concluding that there are resources at risk that the public may value. Two stories resulting from these explorations into the cascading effects of acid rain on terrestrial resources are ideal for effective science communication: the relationship between (1) balsam fir as a popular Christmas tree and habitat for the snowshoe hare, a favorite of wildlife viewers, and (2) white ash because it is used for half of all baseball bats, fine wood products, and musical instruments. Thus, rather than focusing on biological indicators that may only be understood or appreciated by specific stakeholders or experts, this approach extends the analysis to include impacts on FEGS and humans. It also lays the foundation for developing stakeholder-specific narratives, quantitative measures of endpoints, and for conducting demand-based valuations of affected ecosystem services.

## INTRODUCTION

The relevance and applicability of scientific research to the general public is often not effectively communicated. For example, people may not be motivated to act if they learned that the chemical criterion threshold for critical loads (CLs) of atmospheric nitrogen (N) and sulfur (S) deposition had been exceeded. Instead, how would someone proceed if they learned that the availability of quality timber for their favorite Louisville Slugger baseball bats, their beloved balsam fir Christmas trees, and viewing snowshoe hares in nature were compromised by air pollution? Might they contact policy makers and land managers for answers and corrective action? Many of the most compelling stories are linked to human health and mortality (i.e., ozone and particulate matter, Cooter et al. 2013). However, the scope of this work was to communicate compelling stories that reveal how and where humans are inextricably connected to nature (Olson 2009) to foster better stewardship of the valuable natural resources that are at risk from combined N and S deposition.

Combined N and S deposition can directly and indirectly impact the condition and health of forests through what is commonly called acid rain (Bobbink et al. 2010, Bobbink and Hettelingh 2011, Pardo et al. 2011, Greaver et al. 2012). Tree growth often declines and susceptibility to stressors like drought, frost, pest damage, disease, and ozone increases, leading to increased mortality (Cronan and Grigal 1995, DeHayes et al. 1999, Driscoll et al. 2001, McNulty et al. 2005, Fenn et al. 2006, Ouimet et al. 2008, Bobbink and Hettelingh 2011, Pardo et al. 2011). Recognizing the effects of impaired air quality on human health, emission reduction efforts over the last 20 years have resulted in decreases of wet deposition of sulfate (SO_4_^−2^) and oxides of N (NO + NO_2_) by an average of 42–44% and 16–27%, respectively (U.S. EPA 2011). However, additional chemical species of N that are emitted to the air and contribute to total N deposition, such as other oxidized forms and reduced and organic N, are not regulated for the ecological effects caused by total N deposition. Total deposition over 30 kg·ha^−1^·yr^−1^ for S and 20 kg·ha^−1^·yr^−1^ for N still occur in parts of the USA (Zhang et al. 2012, Schwede and Lear 2014). In addition, ammonia (NH_3_) concentrations are increasing in the atmosphere, largely due to agricultural activities (Lehmann et al. 2007, Xing et al. 2012) and modern catalytic converters on vehicles designed to lower carbon and NOx emissions, which reduce NOx to NH_3_ in the process (Kean et al. 2000). These heightened emissions have contributed to increased ammonium deposition, which can indirectly contribute to acidification through nitrification and leaching. Therefore, despite the national trends of reductions in wet NOx-N and S, there are concerns that forest ecosystems continue to be negatively impacted by N and S deposition (U.S. EPA 2009).

The ability of forests to buffer cumulative N and S deposition is often quantified by the use of CLs. A CL is defined as “the quantitative estimate of an exposure to one or more pollutants below which significant harmful effects on specified sensitive elements of the environment are not expected to occur according to present knowledge” (Nilsson and Grennfelt 1988, UN ECE 2004). Using the Simple Mass Balance Model, McNulty et al. (2007) found that terrestrial CLs of acidity were exceeded by current rates of N and S deposition in many of the forests in the northeastern USA and mid-Atlantic, especially in New England, Pennsylvania, and Virginia. Similarly, Duarte et al. (2013) reported that N and S deposition rates exceeded critical acid loads in 45% of 4000 forested plots in the northeastern USA. Therefore, concerns by regulators, land managers, and the scientific community regarding the continued impacts of N and S deposition on forest ecosystems in the USA are supported by recent studies.

Critical load exceedance is used to describe the pollutant loading at which chemical conditions of soils or surface waters negatively impact the biological indicators that are sensitive to them, though rarely are these impacts translated into changes in “ecosystem services.” In other words, the effect of these impacts on humans and their well-being is often not addressed. One reason is that the connections between humans and natural systems, including the connections between biological indicators and human well-being, are complex. Even individuals who are strongly affected by changes in these indicators may not understand or perceive how they are connected to them. The concept of *final* ecosystem services is therefore particularly useful for making the link from CLs to human benefits. Final ecosystem services are defined by Boyd and Banzhaf (2007) as the components of nature that are *directly* enjoyed, consumed, or used to yield human well-being. Applying this concept means that, to understand how humans are affected by changes in biological indicators, we must begin by identifying the final ecosystem services that are affected by these changes, and by specifying the ecological processes that link these ecosystem service impacts to changes in the indicators.

To help identify the final ecosystem services affected by exceedances of terrestrial CL of acidity by N and S deposition, we used terrestrial acidification as an input into the Stressor Module of the STEPS (Stressor–Ecological Production function–final ecosystem Services) Framework (Bell et al. 2017). We used the Final Ecosystem Goods and Services Classification System (FEGS-CS, U.S. EPA) in the Final Ecosystem Services Module to explicitly define a categorization of environmental classes and subclasses (i.e., the landscape on Earth where these FEGS occur) containing the valued components of nature, as well as the beneficiaries (i.e., people or groups interacting with the final ecosystem product) who directly enjoy, consume, or use the components of nature. The FEGS-CS is also useful because it is designed to avoid double counting of ecosystem services, which can be problematic with other classification systems (Nahlik et al. 2012).

Applying the STEPS Framework is by nature a transdisciplinary process, ideally bringing together social and natural scientists, resource managers, and policy makers to identify which FEGS are affected by CL exceedances and to specify the ecological processes that connect these exceedances to ecosystem services. For this analysis, we engaged a transdisciplinary team comprising one economist, an academic ecologist, two policy maker/ecologists, and one public land manager/ecologist. We used the STEPS Framework and evidence from the natural science literature to describe specific “causal chains” (i.e., series of cause-and-effect relationships) associated with two economically, ecologically, and socially (i.e., aesthetically) important acid-sensitive tree species, balsam fir (*Abies balsamea*) and white ash (*Fraxinus americana*). These two tree species are commonly found in northern and northeastern USA hardwood and conifer forests (Duarte et al. 2013).

The STEPS Framework includes a ranking and scoring model to describe the cause-and effect links downstream of the CL exceedance and tie them to the FEGS both quantitatively and qualitatively. Applying this approach to forest ecosystems, we asked: (1) “Are there important ‘chains’ from terrestrial acidification CLs, to ecological response, to human welfare using the FEGS-CS, and if so, could they be used for effective science communication, and (2) do CL exceedances that negatively affect balsam fir and white ash result in declines in forest ecosystem services that can be translated into a number of chains?”

## METHODS

### STEPS framework

The STEPS Framework that was developed for use at the Air Quality and Ecosystem Services workshop (Blett et al. 2016), from which this work originated, consists of three modules to link changes in a biological indicator to a measure of human well-being impacted by the change (Bell et al. 2017). We used the acidification of terrestrial ecosystems to develop the Stressor Module, the Ecological Production Function Module, and Final Ecosystem Service Module to create a series of causal chains that can be used to identify the impact on human well-being. The following sections describe our approach to each of these modules and detail the scoring method used to evaluate the strength of each of our chains.

### Stressor module

#### Species selection.—

While there are many biological indicators in forests for which terrestrial acidification CLs have been developed, we focused our efforts on two acid-sensitive tree species that are well known and appreciated by the general public, that harbor bird and mammal species, and that provide popular downstream products that would be relatable to a broad audience. We selected one conifer (balsam fir) and one broadleaf hardwood (white ash) commonly found in northern and northeastern USA forests (Duarte et al. 2013). We used the species accounts provided in the USDA Forest Service fire effects database (for balsam fir: Uchytil 1991, and for white ash: Griffith 1991) because they provide a review of the range and ecology of the tree species itself, as well as related plant and animal species. We also conducted a more extensive literature review to identify economically and socially important products and FEGS associated with each species.

#### Critical load production function.—

We obtained the N and S CL production functions for balsam fir and white ash from the following sources: the National Critical Load Database developed by the Critical Loads of Atmospheric Deposition Science Committee of the National Atmospheric Deposition Program (http://nadp.sws.uiuc.edu/committees/clad/db/), Battles et al. (2014), Pardo et al. (2011), and Duarte et al. (2013). The base cation/Al ratio is an effective indicator of acidifying deposition in soils that also relates to trees. We used a base cation/Al ratio of 10:1 as a reliable chemical criterion threshold for terrestrial acidification, the ratio at which negative effects of acidification on tree health accelerate (Cronan and Grigal 1995, Duarte et al. 2013). We identified the chains using the ecological production function (EPF) and the FEGS-CS described below.

### Ecological production function and final goods and services modules

**Figure 1.**
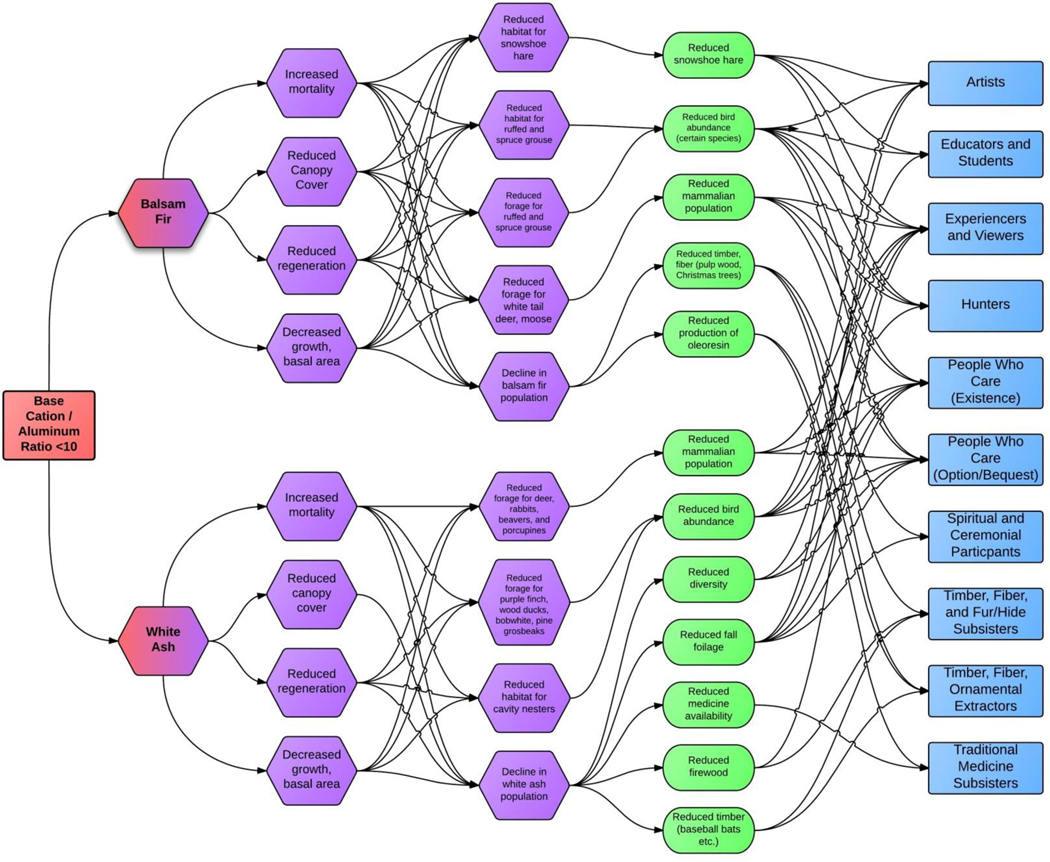
Combined diagram for balsam fir and white ash showing the complexity of all 160 linkages between the FEGS and their beneficiaries for forest ecosystem processes impacted by terrestrial acidification. Ecological production functions are based on the change in growth of balsam fir and white ash. The critical load production function (shown in red), ecological production function (shown in purple), ecological endpoint/FEGS metric (shown in green), and beneficiaries (shown in blue) are represented.

An EPF links changes in a biological indicator (e.g., decreases in white ash biomass) associated with CL exceedance to changes in an ecological endpoint directly used or appreciated by humans (Fig. 1, e.g., changes in fall foliage or reductions in abundance of bird species). In cases where the affected biological indicator was itself an ecological endpoint that is directly used or appreciated by humans (e.g., tree biomass for timber harvesters), no EPF was needed or created.

To complete the causal chains, we used the STEPS Framework with the FEGS-CS to identify specific classes of human beneficiaries who derive final ecosystem services from forests in which balsam fir and white ash are adversely affected. The FEGS-CS defines three main environmental classes with 15 subclasses, and 10 main potential beneficiary classes with 38 subclasses (Landers and Nahlik 2013). The official definition of a FEGS incorporates the environmental class and the beneficiary class, but we use the term “FEGS” hereafter to represent the environmental component used, valued, or appreciated by the beneficiary. The main beneficiaries of FEGS provided by balsam fir and/or white ash would be people who derive direct benefits from three types of natural components: (1) the trees (and materials) themselves, (2) the trees providing or supporting specific animal species’ habitat, and (3) the overall forest ecosystem (i.e., biodiversity). Each of these groups can be linked to specific beneficiary types using the FEGS-CS classes (e.g., 08—Learning) and subclasses (e.g., 0801—Educators and Students).

### Strength of science scores

According to the STEPS Framework (Bell et al. 2017), each module and/or relationship within a module was given a “Strength of Science” (SOS) score based on the literature supporting the link. The SOS is a qualitative assessment of the evidence supporting the chemical criterion (base cation/Al ratio in our case) and CL exceedance that causes changes in the biological indicator (i.e., balsam fir and white ash). The SOS of the effects between two components in the EPF (SOS_E_) is a qualitative assessment of the evidence supporting the relationship. SOS_E_ scores were given values of high = 1, medium = 0.67, and low = 0.33 based on the amount of published literature supporting the relationship (for more information, see Bell et al. 2017). To score and rank the identified relationships, we calculated the SOS for the EPF (SOS_EPF_; Eq. 1) and for the entire chain (SOS_C_; Eq. 2).

(1) $${\text{SOS}}_{\text{EPF}}=\frac{\sum {\text{ SOS }}_{\text{Effect }}}{{\text{ EPF }}_{\text{Length }}}\times \left(1-\frac{1}{M-{\text{EPF}}_{\text{Length }}}\right)$$

(2) $${\text{SOS}}_{\text{C}}=\frac{{\text{SOS}}_{\text{Stressor }}+\left({\text{SOS}}_{\text{EPF}}\times {\text{EPF}}_{\text{Length }}\right)}{{\text{EPF}}_{\text{Length }}+1}$$

The SOS_EPF_ reduces the overall score of the EPF based on the number of components it contains. The equation is meant to account for variability in the importance and intensity of each relationship as more components are added to an EPF. As EPFs get longer, there are more opportunities for components outside those identified to play a role in the responses. The constant M represents the number of components at which the confidence in the connection between the change in the biological indicator and the ecological endpoint is zero. For Eq. 1, M is set to 8.

The SOS_C_ represents the confidence in scientific data, from the change in an indicator due to a stressor to the change in a final ecosystem service. The equation averages the full weight of the SOS_S_ with the diminished value of each SOS_E_ based on the chain length. The two values allow for comparisons and increased awareness of where the impact of the stressor on a biological indicator differs from the awareness of the downstream effects. This value can be used to rank chains according to aspects of ecosystem valuation, beneficiary demand, and/or management objectives.

The Strength of Science–Weakest Link (SOS_WL_) ranks a chain based on the lowest SOS_E_ or SOS_S_ score present in the chain. This is based on the principle that a chain is only as strong as its weakest link and can be used as an alternate measurement of chain strength, highlighting those in which further research is needed.

## RESULTS

As expected, we found that the interrelationships between the FEGS provided by balsam fir, white ash, the forest, and their beneficiaries are complex (Fig. 1) but the effects of acidification were similar on our focal species. Acidification decreases growth and basal area, canopy cover, and regeneration while increasing mortality (Thomas et al. 2010, Duarte et al. 2013, Horn et al. 2015). More specifically, the growth of both species was negatively correlated with CL exceedance, as were the vigor of white ash and crown condition of balsam fir. Similar responses of balsam fir to high N additions have also been reported by McNulty et al. (1996), who found that repeated N deposition resulted in reduced growth and regeneration and increased mortality of the species.

**Figure 2.**
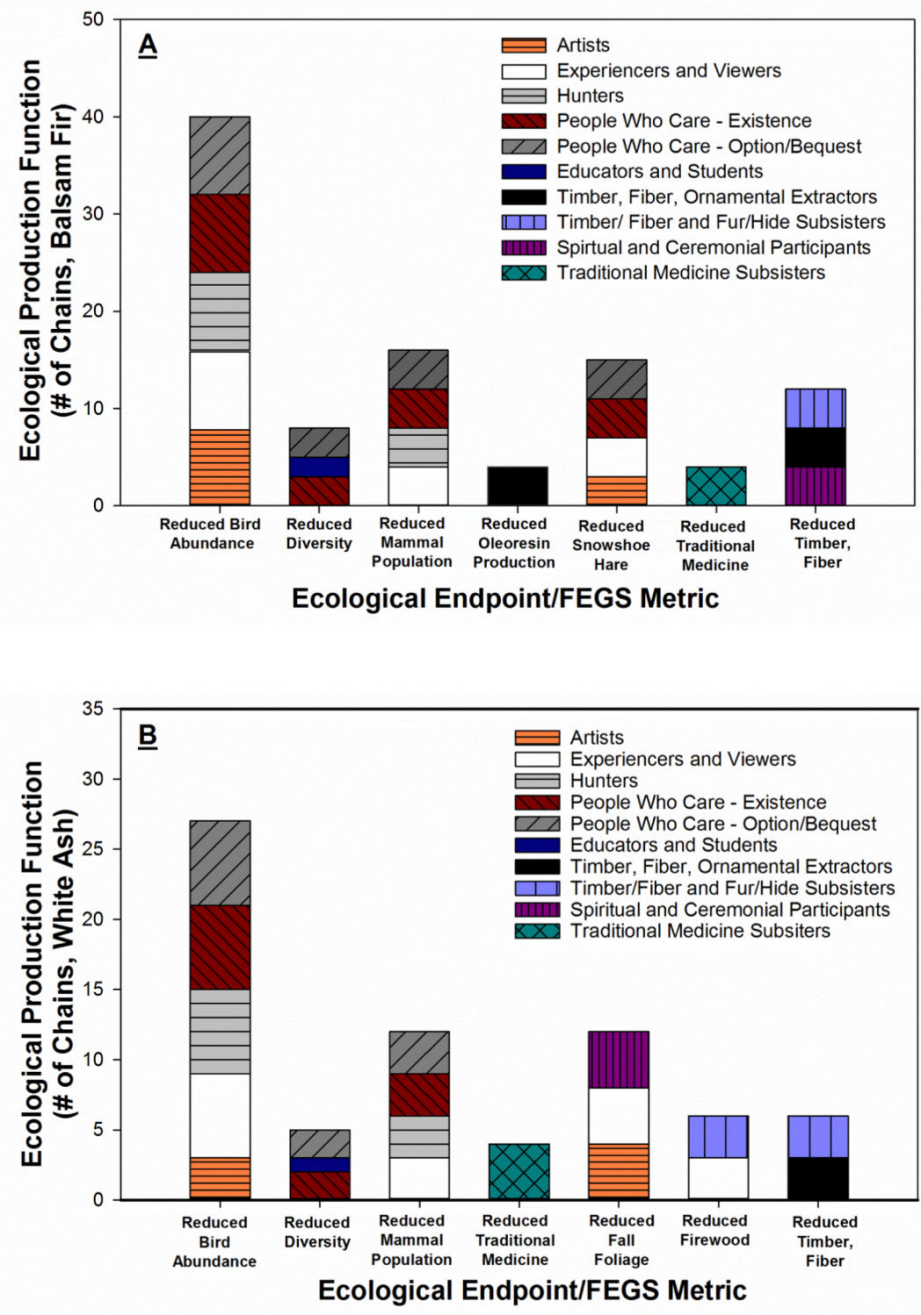
Summary of the number of ecological production functions grouped into FEGS beneficiary classes, for (A) balsam fir, and (B) white ash.

**Figure 3.**
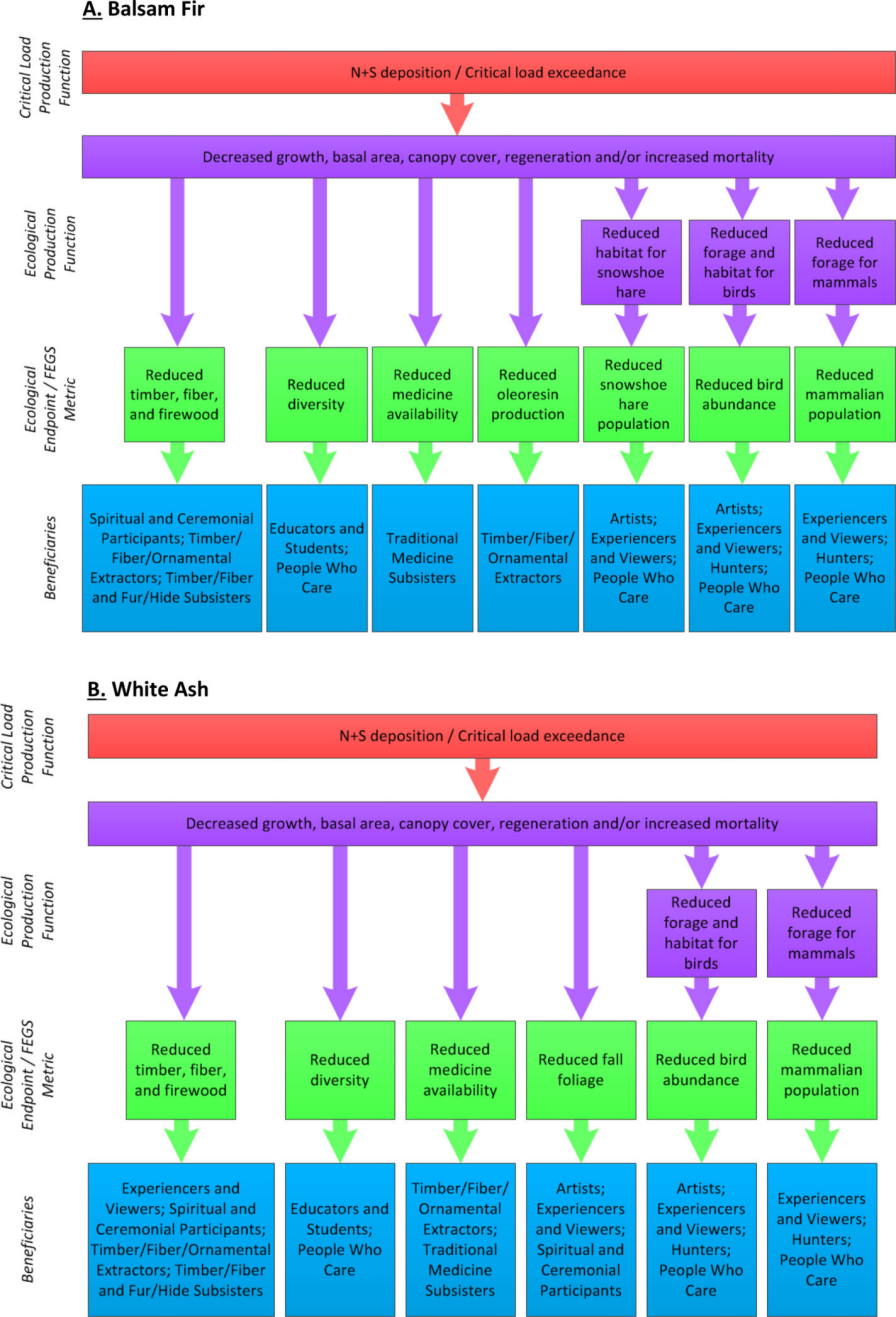
Flow chart summarizing the effects of terrestrial acidification on (A) balsam fir canopy dieback and impacts on the snowshoe hare, and (B) white ash and the associated impacts to multiple FEGS: timber, medicinal/traditional uses, and fall foliage color. The critical load production function (shown in red), ecological production function (shown in purple), ecological endpoint/FEGS metric (shown in green), and beneficiaries (shown in blue) are represented.

Through these changes in our focal species, we identified a total of 160 distinct chains within six beneficiary classes and ten beneficiary subclasses for balsam fir and white ash combined (see Data S1 for the complete table) and the environmental subclass was Forests (21). Balsam fir had 91 chains within six beneficiary classes (Commercial/Industrial [02], Inspirational [07], Learning [08], Nonuse [09], Recreational [06], and Subsistence [05]) and 10 subclasses (Artists [0702], Experiencers and Viewers [0601], Hunters [0603], People Who Care—Existence [0901] and Option/Bequest [0902], Spiritual and Ceremonial Participants [0701], Educators and Students [0801], Timber/Fiber/Ornamental Extractors [0202], Timber/Fiber and Fur/Hide Subsisters [0503], and Traditional Medicine Subsisters [0505], Figs. 2a, 3a). Traditional Medicine Subsisters is a new subclass that will be added to future versions of the FEGS-CS as a result of this work (A. Nahlik, *personal communication*). White ash had 69 chains within the same six beneficiary classes and 10 subclasses as balsam fir but some linked to different FEGS, for example, fall foliage (Figs. 2b, 3b). We identified 12 FEGS and all associated chains resulted in reductions in their ecological endpoint metric (e.g., number of mammals, Data S1). The balsam fir FEGS were the abundance and/or quality of certain bird species, snowshoe hare, other mammal populations, timber/fiber/pulpwood/Christmas trees, and oleoresin production. The white ash FEGS were the abundance and/or quality of birds, mammal populations, fall foliage, diversity, firewood, timber for baseball bats and other fine wood products, and traditional medicine availability. In total, we found 61 one-component chains and 99 two-component chains.

We found high overall SOS_E_ scores for all components of the EPFs because all relationships to balsam fir and white ash were identified based on sufficient scientific evidence in the literature before attempting to describe the chains. This lead to all SOS_WL_ scores having a value of 1.0. The one-component chains had an SOS_EPF_ value of 0.86 and an SOS_C_ value of 0.93, while the two-component chains’ SOS_EPF_ value was 0.83 and the SOS_C_ value was 0.89 (Data S1). Without response curves associated with each of the identified relationships to understand the rate of change of each of the identified relationships, the chains are differentiated by the EPF Length component of the SOS_EPF_ or SOS_C_ scores. This distinguishes between the chains where a CL is directly affecting a FEGS and the longer chains where there are potentially indirect factors outside of the stressor impacting the change in FEGS.

### Balsam fir chains with compelling stories

**Fig. 4.**
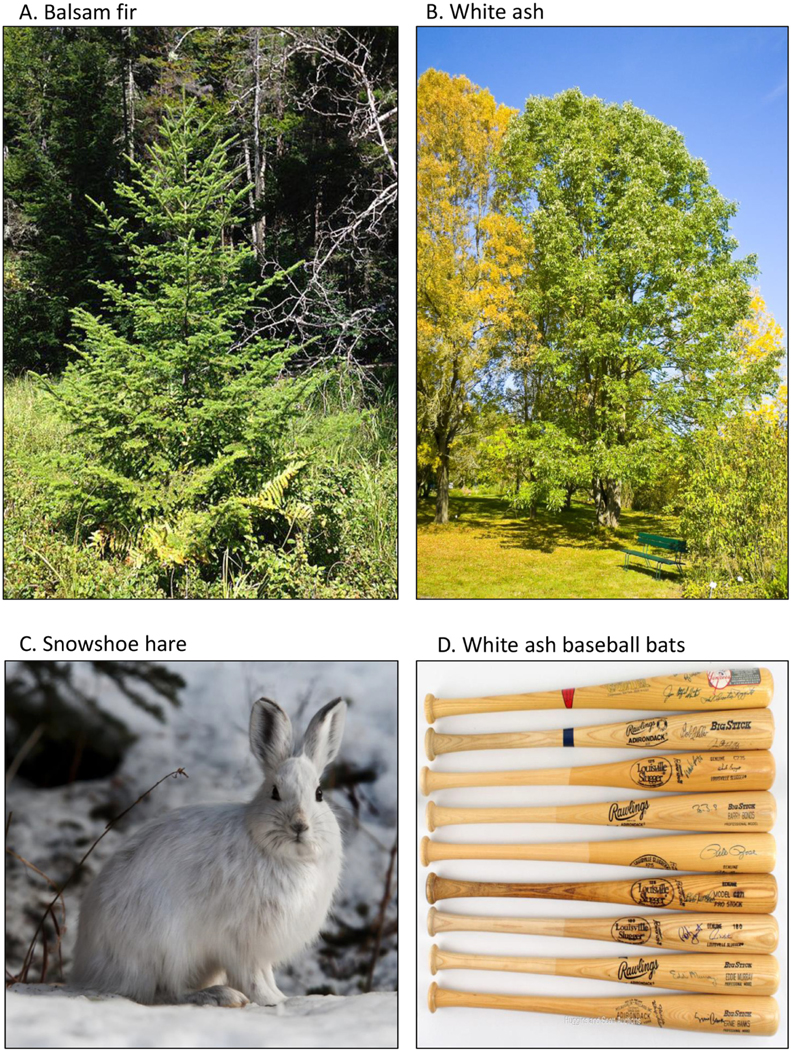
Photographs of (A) balsam fir, (B) white ash, (C) snowshoe hare, and (D) white ash baseball bats with notable autographs. Permission use photographs A, B, and C granted by Creative Commons. Use of and photo credit for photograph D, courtesy of www.hugginsandscott.com.

In addition to its thriving Christmas tree and essential oils industries, and oleoresin production, the balsam fir provides forage and habitat for charismatic mammals (i.e., white-tailed deer, moose, and snowshoe hare) and birds (i.e., ruffed and spruce grouse). The snowshoe hare (*Lepus americanus*), an adored mammal by many, is particularly at risk. As the canopy of the fir decreases due to reduced growth rate and increased crown dieback, the amount of cover available for the snowshoe hare to hide within decreases, increasing predation (Belovsky 1984, Sullivan and Sullivan 1988, Griffin et al. 2005, Creel and Christianson 2008). The loss of forage can also reduce the reproductive rate of the snowshoe hare, leading to a decline in reproductive success (Hik 1995). These stressors act together to diminish local populations impacting the lynx (*Lynx canadensis*), a threatened species for which the snowshoe hare is key prey, and other predators such as wolves and coyotes (Figs. 2a, 3a, 4a, c).

### White ash chains with compelling stories

White ash is considered the most valuable timber tree of all the various ashes for its strength, heavy weight, elasticity, and shock resistance (Schlesinger 1990; Figs. 2b, 3b, 4b, d, and Data S1). It provides habitat and forage for a variety of birds (e.g., spruce and ruffed grouse, purple finch, bobwhite, pine grosbeaks, turkey) and specifically for cavity-nesting birds (e.g., redheaded, red-bellied, and pileated woodpecker), and secondary nesters (i.e., gray squirrel, nuthatches, wood ducks, owls, Runde and Capen 1987, Griffith 1991). Several mammals also depend on white ash (e.g., deer, moose, mice, fox squirrels, rabbits, beavers, and porcupines). It has many traditional medicinal uses, and it is a favorite of those viewing its golden fall foliage. However, we focused on the products that are made from white ash such as baseball bats, musical instruments, fine tools, flooring, and cabinets. We felt the strongest tie was to baseball, America’s pastime, as about half of the bats used in major and minor league baseball are made of this wood. As growth rates and wood quality decline due to the effects of acidification, the availability and potentially the usefulness of white ash for bats may decline as well. Interestingly, two other popular tree species that are used for baseball bats, sugar maple (Acer saccharum) which is gaining popularity and yellow birch (*Betula alleghaniensis*), are also acid-sensitive (Duarte et al. 2013). These three species are preferred for their hardness, tensile strength, elasticity, and weight, each having unique characteristics that suit or enhance an individual player’s batting style. If these species are not readily available, manufacturers may have to shift to different, less desirable species.

## DISCUSSION

Reductions in balsam fir and white ash could be expected on sites where deposition exceeds the CL, which would have cascading effects on ecosystem services. Reduced growth of white ash seedlings under conditions of increased acidity (Chappelka et al. 1988) and reduced survival of both balsam fir and white ash with increasing amounts of N deposition (Horn et al. 2015) have been reported. Applying the STEPS Framework allowed us to successfully and effectively describe how critical load exceedances of terrestrial acidification traveled downstream through EPFs to impact FEGS. Our analysis of how CL exceedances negatively affect balsam fir and white ash revealed 160 important chains linking the decline in the forest to human welfare. For example, if the CL exceedance causes mortality in balsam fir, this can lead to a decline in timber and pulpwood, which would affect timber extractors. We found that this methodology was ideal for quantifying FEGS because, if balsam fir is lost from a forest or region, the number of causal chains associated with that species will be lost over time unless another species takes over its function. This is an underestimate of the number of potential chains as we grouped species responses into animal groups to simplify our analysis. These results can be downscaled to a particular area to identify specific species (and beneficiary subclasses) disrupted by the CL exceedance(s).

Furthermore, Duarte et al. (2013) reported that there are 15 species in addition to balsam fir and white ash that are negatively affected by acidification in the northeastern USA. If these additional 15 species have the same average number of chains as our two focal species (i.e., ∼80 chains), the total number of FEGS affected by terrestrial acidification effects on trees in the northeastern USA is ∼1200. Future work directed at mapping out FEGS for these additional species is needed because this may identify other indirect ways in which forest goods and services are impacted by acid deposition.

Interestingly, a large proportion of the FEGS impacted the same six beneficiary subclasses: Artists, Educators and Students, Experiencers and Viewers, People Who Care (Existence), People Who Care (Option/Bequest), and Spiritual and Ceremonial Participants (Fig. 2). These subclasses are all non-consumptive users that generally exist in specific groups that typically interact with a small area of the environment. Using this information, the next stage is to evaluate the measures of human well-being impacted by these chains to determine the non-consumptive benefits provided by our focal species as well as the forest ecosystem. Although the number of beneficiary classes is not necessarily a measure of the magnitude of the benefits provided, our study suggests that the non-consumptive value may be as or more important to humans than the tangible products derived from the trees and forests. This finding lends support to the work of Louv (2005), who coined the phrase, “nature-deficit disorder,” describing a boost to creativity and health when humans experience nature, and the many consequences of a divorce between humans and nature.

In addition, the SOS scoring system can help discover where more research is needed to better understand the cause-and-effect relationships within an EPF. However, because we began with a literature search and only used the chains with strong scientific evidence, the SOS_C_ scores only slightly differed. By expanding our chains to incorporate undefined reactions in forest shifts, we could have highlighted areas of research needs.

The final way that the number of chains can be expanded is by examining the forest as a whole. Therefore, a method should be developed that scales up the effects of acidification on ecosystem-level processes such as water quality and carbon sequestration, rather than for the species-level effects that were our focus. We did not include forests for detailed analysis because, while there was solid evidence about CLs, there were uncertainty and more speculation about how acidification affects many of these broader and very important ecosystem-level EPFs, for example, due to nitrate and base cation leaching, and episodic stream acidification.

### Challenges to the process

There were several noteworthy challenges identified during our chain development process (reported in Blett et al. 2016). First, there were issues related to *data repetition*. Each biological indicator can have multiple responses to acid deposition that all have the same FEGS ecological endpoint (e.g., increased mortality, reduced regeneration, reduced growth, all leading to total reduced biomass and the FEGS associated with reduced biomass). Because of this, there was a high volume of replicated data since multiple species experienced the same chains, which required streamlining for a useful interpretation. Secondly, there were issues of *forest ecosystem resilience and species redundancy*. There was uncertainty about how to represent FEGS that are not unique to a single species and that could be compensated for by other species in a forest (e.g., carbon sequestration, water quality related to reduced root biomass, or air quality related to reduced canopy vigor or biomass). Also, many different birds and mammals were represented in the chains associated with the uses of the trees, so to what degree is the loss of forage and habitat offered by a specific species replaced by others? If species respond at different rates, is there a threshold for the rate of decline of a species where it will not be replaced? For example, additional research needs to be conducted on the relationship between decreased tree health due to aluminum toxicity and infestation by the emerald ash borer (*Agrilis planipennis*). This pest is a growing problem within the range of the white ash, but it is currently a less-preferred food source than green ash (*Fraxinus pennsylvanica*) and black ash (*F. nigra*; MacFarlane and Meyer 2005). Since both natural and societal systems adapt to and compensate for the loss of a FEGS, there were challenges of appropriately representing and incorporating them in both systems. Thirdly, there were issues of *scale*. We had to consider that there were FEGS offered by the forest as a unit and not just at the watershed, stand, or tree level. For example, forests offer many downstream benefits of clean air and water that had to be represented within the matrix. We reconciled how and where to represent clean air and water in FEGS based on “what is last experienced” by the beneficiary. Fourth, there were issues related to *our current understanding of successional changes in forests as a result of acidification*, which is lacking. Finally, there were *potentially confounding effects of multiple stressors* like ozone on the biological indicator response (e.g., white ash, Chappelka et al. 1988). For example, the typical symptoms of ozone pollution are stippling, reddening, flecking, and bronzing, which are gradually obscured by chlorosis and necrosis in both acid-sensitive and non-acid-sensitive species. Chlorosis and necrosis also are symptoms of acidification; therefore, it can be difficult to disentangle the effects of these stressors where acidification and ozone co-occur.

### Recent differences found in white ash responses

The sensitivities of balsam fir and white ash to the acidifying impacts of N and S deposition identified by Duarte et al. (2013) showed that both species exhibited negative growth in response to exceedances of CLs of acidity. Similar responses of the two species were demonstrated by Horn et al. (2015) who, through a nation-wide assessment, found that the growth of balsam fir and the survival of both balsam fir and white ash were reduced with increasing N deposition. Conversely, Thomas et al. (2010), in their analysis of growth and survival responses in 19 eastern USA states, reported a positive relationship between N deposition and the growth of the two species and the survival of balsam fir. However, despite these apparent inconsistencies of responses, increases in growth with N deposition in one set of studies are not necessarily inconsistent with a decrease in growth following CL exceedance or higher levels of deposition reported in other studies. These differences may be due to a combination of factors including different analysis scales, and the sensitivity of the sites to N deposition causing eutrophic or acidifying conditions for balsam fir and white ash. Further study is needed to harmonize these findings and advance our understanding of these multiple processes that simultaneously operate in any given ecosystem.

### Applicability of chains for use in science communication

The relationships between the balsam fir impact on snowshoe hares and the white ash impact on baseball are two particularly compelling stories that may be helpful for communicating to a broad audience that there are resources at risk about which the general public may care deeply. For many researchers, describing their research or related ecosystem services in these terms may be a relatively new approach (Olson 2009). However, there is no disagreement that billions of dollars around the world are spent researching and managing natural resources for use and enjoyment now and in the future. In a fast-paced world that is increasingly competing for an individual’s attention, the stories that are technically sound, can withstand scientific scrutiny, advance policy, and resonate with the general public are key to people caring about and acting to protect ecosystem services.

## CONCLUSIONS

By focusing on the effects of acidifying N and S deposition on forest systems in the northeastern USA, this work demonstrates how the STEPS Framework, which produces chains to connect critical load exceedances to final ecosystem services and beneficiaries, can provide a useful tool for describing the potentially wide-ranging effects of air pollution on human well-being. By detailing these connections, it may also be a useful framework for evaluating and comparing the implications of potential secondary air quality standards for NO_x_ and SO_x_. Rather than simply focusing on changes in specific biological indicators that may only be understood or appreciated by specific stakeholders or experts, this approach extends the analysis to include impacts on ecosystem services and their human beneficiaries. By identifying links to FEGS, this approach defines policy-impacted endpoints that are accessible to a broader audience. It also lays the foundation for developing quantitative measures of these endpoints and for developing “demand-based” measures of human well-being of the affected ecosystem services.

## Supplementary Material

Sup1

Sup2
