# Supplementary material for: Terrestrial acidification and ecosystem services: effects of acid rain on bunnies, baseball, and Christmas trees
Source: Ecosphere. Author manuscript; Available in PMC 2021 Aug 2. (PMC8327964; doi:10.1002/ecs2.1857)
Supplement: Sup2 [file NIHMS1520033-supplement-Sup2.pdf]

Terrestrial acidification and ecosystem services: Effects of acid rain on bunnies, baseball and Christmas trees

Irina C. Irvine, Tara Greaver, Jennifer Phelan, Robert D. Sabo, and George Van Houtven

Ecosphere

Relationships linking a change in a biological indicators of atmospheric deposition to final ecosystem goods and services.

Irina C. Irvine[1*], Tara Greaver[2], Jennifer Phelan[3], Robert D. Sabo[4], and George Van Houtven[3]

[1]Santa Monica Mountains National Recreation Area, Division of Planning Science and Resource Management, US National Park Service, Thousand Oaks, CA 91360
[2]Office of Research and Development, National Center for Environmental Assessment, US Environmental Protection Agency, Research Triangle Park, NC 27709
[3]RTI International, Research Triangle Park, NC 27709
[4]Currently an Oak Ridge Institute for Science and Education (ORISE) Fellow, Office of Research and Development, National Climate Assessment Global Change Impacts and Adaptations, Environmental Protection Agency, Crystal City, VA 22202

DataS1.xlsx

'DataS1.xlsx' lists all of the 160 chains developed to link change in a biological indicator due to exceedance of a critical load to an ecological component that is a final ecosystem service. The table below provides descriptions of each of the columns. This data set can be used to sort by your interested field to evaluate the relationships among the chains and the beneficiary categories. Identifying places within the table where additional stressors are acting will allow us to expand the analysis to evaluate where synergistic effects may occur. It can also allow a user to sort by the type of beneficiary they are interested in to determine if the indicators exist in their area and where management actions can take place.

**Module**	**Column Header**	**Description**
	Ecological Effect	The broad consequence of nitrogen and/or acid deposition on an ecosystem to be evaluated
Site Information	Region	Regional designation of where critical load is applicable
Site Information	Ecosystem	Ecosystem type in which critical load is applicable based on EPA designated Ecosystem Level I, II, and III

Stressor	Chemical Criterion	Soil (e.g., soil solution Bc/Al ratio or % base saturation) or surface water chemistry (e.g., ANC or pH) that links the critical load of atmospheric deposition to the biological receptor of interest. Deposition alters the chemical criterion, and the critical load of a system is the point where deposition levels result in the chemical criterion being equal to the critical limit. Chemical criterion are commonly used in models to estimate aquatic and terrestrial critical loads of acidity. If species change has been measured to correlate with a deposition gradient, the deposition itself is the chemical criterion.
Stressor	Chemical Threshold	The threshold or value of the chemical criterion beyond which the biological receptor of interest is negatively impacted (e.g., 20% base saturation, ANC of 50 μeq/L). The critical load of a system is the point where deposition levels result in the chemical criterion being equal to the critical limit.
Stressor	Biological Indicator	A biological species or group of species whose function, population, or status can reveal the qualitative status of the environment, and can therefore be used to monitor the health of an environment or ecosystem.
Stressor	Critical Load (CL; in kg/ha)	The amount of atmospheric deposition above which adverse effects begin to affect the biological indicator
Stressor	Low	If the critical load has been established as a range; the low value of the range.
Stressor	High	If the critical load has been established as a range; the high value of the range.
Stressor	SOS_S	Strength of science of the calculated critical load
Stressor	CL Reference	Scientific publication used to verify SOS_S
Stressor	Change in Biological Indicator (Component #1)	The effect on the ecosystem component due to exceedance of the critical load or the chemical criterion. The change in biological indicator acts as the first component of the Ecological Production Function

Ecological Production Function	SOS – Effect # (SOS_E)	Strength of Science explaining how the proceeding ecological components causes a change in the proceeding ecological component. Values are High = 1, Medium = 0.67, Low= 0.33
Ecological Production Function	Ref #	Scientific publication(s) linking the change of $Component_i$ to $Component_{i+1}$
Ecological Production Function	Component #	Ecosystem component influenced by a change in the ecosystem component before it.
Ecological Production Function	Ecological Endpoint	Ecological component that provides a service or is valued by humans. This is the same as the last link in the chain, but allows for chains to be categorized due to different chain lengths.
Final Ecosystem Services	Beneficiary sub-classes (direct FEGS user)	FEGS-CS Beneficiary Sub-Class: beneficiary that directly uses the last thing in environment directly used by humans.
Final Ecosystem Services	Beneficiary Classes	FEGS-CS Beneficiary Class
Final Ecosystem Services	FEGS Metric	The measurement taken to classify the Ecological Endpoint in a form relevant to the beneficiary sub-class
Strength of Science	Chain Length	Number of links within the Ecological Production Function
Strength of Science	SOS_{EPF}	Calculation of EPF using number of components in the chain and the average score of the chain, Equation 1
Strength of Science	SOS_{WL}	Value of the weakest SOS_E or SOS_S score in the chain
Strength of Science	SOS_C	Calculation using the SOS_E and SOS_S scores to define the confidence in scientific knowledge of the relationships; Equation 2
